# Zombie Days: How Schizophrenia Almost Made Me a Card-Carrying Member of the Undead

**DOI:** 10.1093/schizbullopen/sgad034

**Published:** 2023-12-10

**Authors:** Susan Weiner

When I had my first psychotic episode I was in graduate school. Unfortunately, under the influence of psychosis, I withdrew from my academic program, broke up with all my family and friends, and disappeared into the inner city. I believed I was called on to live as a spy on the frontlines of a psychological war that required my undivided time and attention and isolation from my former life. I believed this was a war fought with minds rather than conventional weapons such as tanks and artillery. Giving in to what I assumed were military instructions, I lived a covert life in a psychotic state for 7 whole months, long enough to develop a florid fantasy of a secret guerilla insurrection that sought to liberate my country from an apocalyptic plot furthered by shadowy figures who belonged mostly in a horror movie. For finances, I lived on tuition money that had been allocated to me but not spent on university. Thousands of dollars can go far.

What interests me is my lack of insight into the illness. I completely accepted astonishing propositions such as I was going to become a zombie. If these propositions occurred to me, then I accepted them uncritically as one would a geometry proof that displayed its reasoning step by step. If I thought it, I believed it almost without another word. I never ever questioned my suppositions. With no one to point out the absurdities of my conclusions, especially not myself, I built up a frightening world thought by thought as if putting together data to achieve a synthesis. And I believed the most egregious things despite the fact that I had once had a fellowship to study abroad and ordinarily displayed reasonably good critical skills in life.

Although I had just come from graduate school most of my delusions were developed by listening to what I thought was the radio speaking to me. Some of my craziest ideas even came about because of the physical symptoms involved with a psychiatric illness. I include under this category what I assume was a panic attack that at the time I believed was a heart attack. Also, I often had a tingling sensation that proceeded from my forehead in a line across the part of my hair down to the back of my skull. Sometimes I had abdominal cramps that troubled my tummy and my mind. To me, these physical symptoms appeared to come out of nowhere. Surprisingly, I never considered seeing a doctor as if something physical were seriously wrong. Instead, right away I assumed these phenomena came from external sources discharged into my body, not from my body crumbling under the weight of psychosis.

For reasons that remain unclear to me, I immediately attributed all physical symptoms to a source that beamed rays into me. Like a 1950s TV movie, I thought they came from an invisible, advanced technological system of which I’d been previously unaware. It was like a Star Trek episode where Spock displays telepathic powers just by pinching you on the neck. In some (or in all) ways I had completely suspended disbelief as if my new life were a book of frantic stories I had to make sense of or die. Although I’d lived in a world of facts all my life, fiction completely overcame my consciousness and perception. I’m completely baffled that I never thought of visiting a doctor, even after I thought I had a heart attack. One night I lay down in bed to sleep when my heart suddenly seized up and felt like it was being crushed. I was terrified. This is a heart attack, I thought. I’m having a heart attack. But amazingly, I never thought to call for an ambulance or call my father who was a doctor. Immediately I recognized with blinding insight that I was the victim of a new technology that could reach into your body at will and manipulate the functioning of your vital organs. Because it didn’t happen often, I surmised this was a new technology being tested on me. I had reasons for everything. And reasons for nothing.

Especially notable to me were times I thought I was being tortured or mentally twisted and warped. I attributed these experiences to malicious, wicked actors. In my head, I thought evil military sources were persecuting me as they tried to discover my whereabouts in the country and drive me crazy. Ironically, at all times I was very afraid incidents of what I thought of as torture would drive me mad. Never in the world did it occur to me that my brain was malfunctioning. I was terrified of just that very thing happening. The absurdity or irony of it all is remarkable. I was mad but I believed I was sane, and I was terrified I would actually go mad without warning. Who could dream these things up? Who could save me?

It staggers me that the mind can create all sorts of psychological (or even physical) symptoms that are normally the province of hallucinogenic drugs without insight into the source of the problem. Physical experiences had a deep influence on the track of my delusions and caused me to dig down further to function as a clandestine operator in my new world. In my mind, the physical buttressed my belief that I was a spy on a dangerous mission. People wanted to harm and/or torture me. Of course, I believed this was because I posed an existential threat to a kingdom of evil that I sought to overthrow.

One afternoon, deep into the months of psychosis, I was lying on a couch with my eyes closed. Suddenly in the dark, right in front of me, as if my eyes were completely wide open, a bright and colorful animated figure rushed from left to right before me. Then it went the other way. Back and forth, bright and shining like a comet lighting up the night sky. Then without warning all sorts of animated creatures began to rush back and forth, back and forth, up and down, left to right, right to left, top to bottom, speeding at such a degree that it was as if I were experiencing a Bugs Bunny comic on methamphetamines. The images of cartoons were everywhere. But they were too bright.

I can’t tell you how much they hurt my eyes and how disorienting the experience was. The images were so distressingly intense. They were dazzling and neon-colored, and I really feared I might go blind from their brightness. It was like looking directly into the sun. It felt like a violent assault on my retina. And I couldn’t make it stop. It was like a Haight-Ashbury circus gone mad. It was agonizing painful and I waited for it to dissipate but instead, it grew worse. It seemed to go on forever. The images continued until I opened my eyes in desperation. For over two weeks I couldn’t close my eyes before sleep until I basically passed out from exhaustion. Then one day as surprisingly as it had come on, the sensation receded and was gone.

Symptoms such as this only served to reinforce my delusional belief system. Instead of thinking there was something indelibly wrong with my brain or my eyes like any normal human being, I reflected on the phenomenon and assumed I was being tortured. I had been hearing low murmuring in the background sometimes as if people were talking in another room. Both experiences were consistent with my imaginary world where a malevolent figure sought to harm me. I’d like to note again that madness was a constant threat to me and always a tool on the side of evil; never in my own mind did I realize I might be sick. If you had suggested that to me, I would’ve assumed you were the enemy right away. I would’ve believed you meant to do me in. Later, in a psychiatric hospital, a doctor did try to reason with me and suggested I was not quite well. I thought he was with the CIA and I hated him unconditionally. Fortunately, he later became my psychiatrist and friend.

Instead of running to an ophthalmologist, I concluded that my belief system (though highly unbelievable even to me who didn’t question it) was correct. There was an evil entity who was bent on destroying the country and me. He had tried to kill me with a heart attack, and now he had attacked my vision and was causing me to hear disturbing voices babbling like a low-volume radio. Each new painful experience reinforced my belief that a clandestine war in the United States was being fought on desperate lines. Painful incidents proved to be a real-life indication that I was engaged in a terrible struggle of good against evil. One incident reinforced another. Each incident wasn’t just an exception. To me, it was an obvious case of struggle that built from one horror to another, and I realized I was on the frontlines of a type of French Resistance but worse. I hardened myself to struggle. Although I was afraid, I was not afraid. I was curiously courageous in sickness. If only I were sane, I would’ve run as fast as I could to the first and most reputable doctor I could find.

Another time, I was on a road trip across the country trying to stay ahead of what I thought were those pesky, malicious authorities pursuing me. As I was driving through the state of Utah I came across a scenic overview with a pass to pull over and take in a particularly striking canyon. I decided on the spur of the moment to take a break and take advantage of the natural beauty of the American West. I pulled my car over from the roadway and hiked in a little to a large flat-topped cliff where I sat down to appreciate the colors and rock formation up and across the hills. But pretty quickly I noticed something disturbing. I could see that the view was striking. I could see that it was what we might call magnificent, but I didn’t have any feelings corresponding to that recognition. It brought me no joy to look at the wildly beautiful landscape. I saw that the rocks were extraordinary and wonderfully colored in shades of orange, peach, and red. But in response to that, I felt nothing. I had no emotion to correlate with what I perceived. I felt absolutely zero about how gorgeous the spiraling colors of the canyon were. I was horrified. I was sick at heart. What was wrong with my mind or my very being? I had no idea I was experiencing anhedonia. I’d never even heard of anhedonia.

Running it over in my mind I came to the very quick conclusion that the enemy was using mind control to turn me into a zombie. A real-life, brain-eating, drooling, vicious zombie. Because everyone knows a zombie has no feelings and here was direct evidence that my emotions were disentangled from my very being. Now, I was a PhD candidate at my university. And yet I found it perfectly logical and reasonable that an evil presence would use some kind of unthinkable, invisible, mind ray to create monsters whose only province for me before had been on TV. I was literally petrified. I understood that only a zombie wouldn’t feel the grandeur of beauty and I gave in to the delusion that there existed monsters who were used as top-of-the-line assassins in order to threaten a population. I thought if I weren’t careful, I would soon be a monster too. I was deeply unsettled, and distressed. I definitely didn’t want to be a zombie assassin.

Unfortunately, sometime later, I was up late at night in a town on the California coast. I opened my seedy motel curtains to see a dangerous and threatening-looking man shuffling down the street. And there it was. I could see it for myself. A real-life zombie in the flesh. The real horror movie monster unleashed in the world. He moved like a shuffling creature in the middle of the road, eschewing possible cars; he wore tattered, loose clothing; he hunched over as he shuffled as if he were looking for someone or something. Before I knew it, I had concluded that he was out hunting me. It came over me as a terrible realization like a bolt of lightning. I was agonizingly close to being found out and physically, not just mentally, tormented.

In reality, it was just another example of events that possessed me and led me to infer facts about phenomena (or people) as a response to a seemingly obvious truth that was anything but reality-based. Trembling, I hid behind the curtains and locked my door up doubly tight. Here, I thought, was confirmation of my belief that I thought of as a new world of reality. This was an unalterable fact that affirmed my belief and confirmed that I was correct in all my thinking. I was reality testing in my own way and the threats to my existence such as heart attacks, blinding cartoon images, a distinct absence of feeling, and terrifying creatures who only looked on the surface like run-down, sloppy alcoholics proved to me that all my suppositions were true. The more illogical, the more reasonable my thoughts seemed. The more outrageous the event, the more I used it to further and buttress my crazy, paranoid fantasies.

Perhaps you may wonder why people who go insane are often fixated on zombies, robots, or aliens. There is a very simple answer in my case. Recall the events I have recounted. Although I didn’t know I was insane, I knew I was experiencing terrible deviations from ordinary life. Blinding, burning-bright cartoon figures had never troubled me before. If I saw a pretty painting or landscape I usually had a pleasing aesthetic reaction that was deep, meaningful, and intense. It was a shock to lose this foundation of emotion that is so common to us all. I understood that what was happening to me was unusual and not shared by most of the population. Who could I trust but my imaginary friends who gathered (at least in my head) to protect me? To me anyone was a creature of the evil regime so I desperately sought to protect myself by uttering ritual incantations meant to stymie invisible attacks against my mind.

This had never been my experience of life before. It was terribly disorienting. The hardest things I had to cope with were exams and papers written to impress professors who wrote insightful, world-renowned books. My only enemies were catty girls in secondary school. I knew something unsettling was happening to me. I knew that my humanity was unraveling. The alienation of my experience led me to consider outrageous scenarios. I was wholly alone in my perception and understanding. The alienation and distance from my past humanity were striking. I knew it was there. I could feel the dismay and the struggle to communicate with others. It’s a reality that lends itself to the monstrous and the alterity of robots or even aliens. What else could explain the extremes of perception I now experience on a daily basis?

All the torture I feared were incidents on the way to becoming less than a human being. I knew I was no longer the same sort of person I had been in university. This was an obvious conclusion that was actually true. And I definitely feared the decay of what humanity remained to me. Obviously, robots, aliens, and monsters had much more in common in the popular imagination than what I felt: stunted emotion, strange and unwholesome phenomena that were unique in my consciousness, and a belief system that defied logic but seemed so obviously true. I feared what loss each new day might bring. None of this was directly conscious. But it haunted me nonetheless. I was haunted by evil. There was a loss I felt deep in my core but couldn’t have articulated at the time.

And what amazes me most is the lack of insight I showed toward psychosis. Just as I’d laugh at you now if you insinuated I was a monster in the making, I held on to that certainty when sick with a bulldog’s grasp of intuition. In fact, it was partially my intuition that was to blame. It was on fire with new ideas, unbelievable extremes, and a feeling that only I (and a sacred few) really understood the nature of reality as it existed for my country and me. My intuition practically glowed with the truth. If I had a realization such as I was being threatened with becoming a zombie or someone was beaming evil images into my head, I accepted it uncritically with my whole heart and soul. My intuition as it stood was deeply wounded but it seemed to me then the only thing that was whole. When rational, intuition has given us the great steps of renowned scientists. But when sick and deformed, it’s a dangerous way to live and one that almost killed me. That it didn’t is thanks to my first psychiatrist who came to see me in the psychiatric ward where I had fortunately landed. The well-respected Wayne S. Fenton, MD treated me and introduced me to the newest medication of the day, Risperidone. And in doing so, he saved me from all the zombies and evil tyrants who had taken over my life in dreams.

